# Treatment of *Staphylococcus aureus*-infected diabetic wounds by melatonin loaded nanocarriers

**DOI:** 10.1186/s13568-025-01854-0

**Published:** 2025-03-15

**Authors:** Alyaa Farid, Ayah Mohsen, Bassant Nasser, Habiba Alaa, Mariam Abdelaziz, Maryam Mustafa, Mustafa Mansour, Nourhan Adel, Salma Magdy, Salma Mohsen, Samah Adel, Sarah Ibrahim, Shaymaa Abdel-Rahman, Sohaila Mohamed, Yomna El-Karamany

**Affiliations:** 1 Biotechnology Department, Faculty of Science, Cairo University, Giza, Egypt; 2 Biochemistry Department, Faculty of Science, Cairo University, Giza, Egypt; 3 Biotechnology/Biomolecular chemistry program, Faculty of Science, Cairo University, Giza, Egypt

**Keywords:** Diabetes mellitus, Melatonin, Chitosan, Lecithin, *Staphylococcus aureus*

## Abstract

One of the complication of diabetes mellitus is chronic wounds. The healing of wounds in diabetic patients is retarded by the elevation in the pro-inflammatory cytokines secretion and free radicles accumulation. Wound management in diabetic patients requires preventing bacterial biofilm development. Due to the wound healing activity of chitosan (CS), lecithin (Le) and melatonin (M), the present study aimed to load melatonin on CS/Le NPs and examine their effect on diabetic wounds infected with *Staphylococcus aureus*. Melatonin loaded chitosan/lecithin nanoparticles (M-CS/Le NPs) were physically characterized and their antioxidant, anti-inflammatory and antimicrobial activities were examined in vitro. Male Sprague Dawley rats included two division (non-diabetic and diabetic) which were further divided in nine groups. Diabetes induction and follow up throughout the experimental period was confirmed by measuring the levels of fructosamine and blood glucose. Full-thickness wounds was induced in both non-diabetic and diabetic animals followed by infection with *Staphylococcus aureus* according to the experimental design. The wound healing effect of M-CS/Le NPs was evaluated through measurements of the oxidative stress, inflammatory cytokines and apoptotic proteins. Our results showed the anti-microbial, free radical scavenging and hemolysis inhibition effects of M-CS/Le NPs in vitro. Moreover, the preparation of M-CS/Le NPs decreased the dose of used melatonin (when compared to free melatonin). M-CS/Le NPs significantly decreased the wound area percent in treated infected wounds of both non-diabetic and diabetic rats more than free melatonin or unloaded CS/Le NPs. In conclusion, M-CS/Le NPs promoted the wound healing in *Staphylococcus aureus*-infected wounds in diabetic rats.

## Keypoints

1. M-CS/Le NPs showed the anti-microbial, free radical scavenging and hemolysis inhibition effects.
2. M-CS/Le NPs promoted the wound healing in *Staphylococcus aureus*-infected wounds in diabetic rats.
3. M-CS/Le NPs decreased the dose of used melatonin in wound healing.

## Introduction

Diabetes mellitus (DM) is among the most prevalent and serious health problems of the twenty-first century (Casqueiro et al. 2017). According to Casqueiro et al. (2017), there will be about three million people with diabetes by 2025. Egypt is among the top 10 nations in the world for the number of diabetic patients. Over 86,478 persons in Egypt die from diabetes-related causes each year, accounting for 16% of the country’s adult population aged 20 to 79 years old. According to Uppu and Parinandi (2008), there are numerous major problems associated with diabetes, such as obesity, renal disease, cardiovascular disease, inflammation and immunity, and cerebrovascular disease.

Numerous factors may hinder the healing of wounds. There are two types of variables that affect the repair process: systemic and local. Systemic variables, affect a person’s overall health and, consequently, his or her ability to heal. Examples of these include smoking, alcohol abuse, obesity, malnutrition, and chronic diseases like diabetes mellitus. Local variables, on the other hand, directly affect the features of the wound, such as its depth, location, and infection (Guo and DiPietro 2010). Non-healing open wounds are defined as those that take a reasonable amount of time to heal, while chronic wounds are defined as wounds that cannot heal beyond 12 weeks (Sheehan et al. 2003). Although diabetic wounds begin as acute wounds, they eventually undergo numerous complications and delays in the healing process. This prevents the normal healing process for acute ulcers. The healing process of wounds involves a number of growth hormones, cytokines and proteases. Diabetic patients’ ability to heal wounds is impacted by a number of factors, including high blood sugar levels, long-term inflammatory response, a lack of oxygen, neuropathy, and circulatory abnormalities (Baltzis et al. 2014).

Wound healing turns into a complex and dynamic process when the integrity of tissue is compromised. According to Baltzis et al. (2014), there are four phases of wound healing that partly overlay: hemostasis, inflammatory stage, tissue proliferation, and remodeling stage. The 1st phase, known as hemostasis, requires platelets. They are triggered, clump together, and stick to the injured tissue by the aid of collagen (Pradhan et al. 2009). According to Blakytny and Jude (2006), fibrinogen is transformed into fibrin during activation, resulting in the synthesis of blood clot and fibrin-containing scaffold. Activated platelets generate growth factors like transforming growth factor (TGF)-β, which promote the healing of tissue, as well as proteins that enable neutrophils and monocytes recruitment (Falanga 2005). As soon as inflammatory cells reach the wound site, the 2nd phase (inflammation) starts. Neutrophils are recruited to the injured tissue upon the activation of adhesion molecules on the blood vessels endothelial cells. Following this, the neutrophils move farther into the connective tissue space (diapedesis) via the endothelial cell gaps or the burst vasculature (Falanga 2005; Dinh et al. 2012). Neutrophils are very important in tissue regeneration and prevention of infection. Neutrophils also contribute to wound healing by generating growth hormones that promote proliferation of cells and proteases that degrade the extracellular matrix (Dovi 2004). In addition, they generate many pro-inflammatory mediators including cytokines (Koh and DiPietro 2011). T-lymphocytes appear to play a modulatory effect in tissue remodelling and infiltrate the wound site during the late inflammatory phase. After degranulation, mast cells release a variety of cytokines that draw neutrophils and proteinases that degrade the extracellular matrix (Egozi et al. 2003; Weller et al. 2006).

When the inflammation goes down and the macrophages change into a different activated/anti-inflammatory type, the 3rd phase (proliferation) starts. According to Koh and DiPietro (2011), anti-inflammatory macrophages generate many anti-inflammatory facilitators, proteinases’ inhibitors, and growth factors that promote protein synthesis and cell division. The temporary matrix is being substituted by the granulation tissue. Fibroblasts undergo stimulation by macrophages’ released growth factors and transport to the injured tissue; where they proliferate and secrete collagen. Fibroblasts’ growth is continued by angiogenesis. Newly formed blood capillaries must emerge from a current capillary system in order to supply nutrients and O_2_ to the quickly proliferating fibroblasts inside the healing area (Falanga 2005). The remodeling phase starts two to three weeks after the initial injury, during which time granulation tissue gradually transforms into mature scar tissue. When the percentage of blood vessels formation drops, collagen is rearranged. Matrix metalloproteinases activity primarily maintains the balance between the synthesis of new collagen and the degradation of old one throughout the remodeling phase (Falanga 2005; Pradhan et al. 2009).

Normally, skin surface has different types of bacteria that can enter the tissues underneath once the skin gets injured. Biofilms consist of intricate clusters of accumulated bacteria embedded in secreted polysaccharides matrix that is released by the bacteria itself in the infected wound (Edwards and Harding 2004). Developed biofilm provide protected microbial environments and show greater resistance to the antibiotic action. Due to the increased synthesis of inflammatory cytokines like TNF-α and IL-1β, both bacteria and endotoxins have the ability to prolong the inflammatory phase leading to non-healing chronic wound if untreated. The prolonged inflammation also causes an increase in matrix proteinases and a decrease in protease inhibitors production leading to the degradation of the extracellular matrix. In chronic wounds, this change in protease equilibrium could lead to the destruction of growth factors (Edwards and Harding 2004; Menke et al. 2007). The most common bacterial species in infected wound are *Pseudomonas aeruginosa* and *Staphylococcus aureus* (Edwards and Harding 2004; Davis et al. 2008). Several chronic wounds are not healed because of the formation of bacterial biofilms that shield the pathogen from neutrophils’ phagocytosis. This process is account for the ineffectiveness of antibiotics as a wound care strategy (Bjarnsholt et al. 2008; Hardman and Ashcroft 2008).

Melatonin, which has been known for its antioxidant capability, is a hormone that is identified for decreasing multiple indicators of inflammatory response. It accomplishes this by preventing nuclear factor-kappa B (NF-κB) from translocating to the nucleus and attaching itself to the DNA molecule. This prevents the production of multiple pro-inflammatory cytokines. (Reiter et al. 2000). Furthermore, it has been demonstrated that melatonin decreases the oxidative stress by preventing the synthesis of adhesion molecules, which encourage leukocyte adherence to the endothelial cells (Rodríguez et al. 2007).

Melatonin is known for its anti-inflammatory activity in circumstances when immune reactions exceed the normal levels (Carrillo-Vico et al. 2013). For example, melatonin has been shown to be effective during the inflammatory stage of diabetic wound healing (Mirza et al. 2014). It has been demonstrated that melatonin reduces the production of an array of cytokines that promote inflammation (Gitto et al. 2012), the oxidative stress and resulting damage to tissues resulting from free radicals accumulation that lead to a reduced enrollment of polymorphonuclear cells to the tissue (Carrillo-Vico et al. 2013). According to Liu et al. (2020), melatonin promoted diabetic wound healing by raising the expression of the Phosphatase and Tensin Homologue (PTEN) gene and reducing the Protein Kinase T (AKT) phosphorylation, which reduces the infiltration of neutrophils. According to de Souza et al. (2022), topical melatonin treatment improved skin healing in rats with diabetes by reducing inflammation and encouraging an early rise in monocytes and T cells at the wound site. Accelerated cutaneous wound contraction, early collagen fiber maturation, increased accumulation of collagen, and accelerated development of scar connective tissues were all promoted by melatonin.

On the other hand, there are several obstacles related to the physicochemical properties of melatonin that make it unsuitable for use as a medicinal agent. Melatonin has a low solubility in water, a weak dissolving rate, and a high susceptibility for oxidation (Hafner et al. 2009; Kumar Yadav et al. 2017). Melatonin can be used at therapeutic dosages when it is loaded on nanoparticles, which boost the molecule’s photostability and inhibit its breakdown (Tursilli et al. 2006).

According to Sahana and Rekha (2018), biopolymers are organic molecules produced by living creatures and are a common class of biomaterial utilized as nanocarriers. Chitosan (CS), a deacetylated and active form of chitin, has been utilized in medicinal products since it has many characteristics that can facilitate this procedure. Chitosan has been shown to have beneficial effects on tissue regeneration and hemostasis (Ahmed and Ikram 2016), to stimulate fibroblast development (Ueno et al. 2001), and to have antimicrobial and antifungal properties (Raafat et al. 2008). Furthermore, chitosan is a biodegradable and biocompatible polymer that has mucoadhesive qualities (Andreani et al. 2015; Biranje et al. 2019). Lecithin (Le), is yellow/brownish fatty compounds that is present in the tissues of both plants and animals. It is used to describe a variety of amphiphilic substances that are used to smooth food textures, emulsify, homogenize mixtures of liquids, and repel sticking products. These substances also attract both water and fatty substances. Soy lecithin aids in the healing of damaged tissue. In addition to its preventive properties, soy lecithin keeps tissues hydrated and promotes skin healing (Ferreira et al. 2012).

Therefore, CS and Le were used in the production of chitosan/lecithin nanoparticles (CS/Le NPs) due to their combined characters that aided in wound healing. The study aimed to load melatonin on CS/Le NPs and examine their effect on diabetic wounds infected with *Staphylococcus aureus*. Melatonin loaded chitosan/lecithin nanoparticles (M-CS/Le NPs) were physically characterized and their anti-microbial, anti-oxidant, anti-inflammatory activities were examined in vitro and in vivo. The wound healing effect of M-CS/Le NPs was evaluated through measurements of the oxidative stress, inflammatory cytokines and apoptotic proteins in skin tissue homogenates. Diabetes induction and follow up throughout the experimental period was confirmed by measuring the levels of fructosamine and blood sugar.

## Materials and methods

### Materials

Chitosan (CS) (low M.wt, more than 75% deacetylated), melatonin (M) and streptozotocin (STZ) were obtained from Sigma-aldrich, UK. Lecithin (Le) (lipoid S45) was obtained from Lipoid GmbH, Germany.

### Preparation of melatonin loaded-chitosan/lecithin nanoparticles (M-CS/Le NPs)

The CS/Le ratio was 1:1, and the procedure followed was in line with Lopes Rocha Correa et al. (2020). Approximately, chitosan (10 mg/ml) was dissolved in HCl (0.275 N). Five hundred µL of the formed CS solution was combined with 40 mL of distilled H_2_O, and the volume was completed to 46 ml. After dissolving lecithin in absolute ethanol to a concentration of 1.25 mg/ml, melatonin was added (5 mg/mL). Lecithin-melatonin ethanolic solution (4 ml) was added to 46 mL of chitosan solution using a syringe with an inner diameter of 0.75 mm. Ultrapure water was used in place of melatonin while making unloaded CS/Le nanoparticles. To create powdered nanoparticles, the generated M-CS/Le NPs were freeze-dried. Zeta potential was utilized to identify the surface charge of the produced M-CS/Le NPs during the physical characterization process. Dynamic light scattering (DLS) was utilized to determine the size of the nanoparticles. The particle size were measured using a transmission electron microscope (TEM). The physical characterization (size and surface charge) was performed on zero, 15, 30, 60 and 120 days after nanoparticles preparation.

### Measurement of melatonin release (%) from M-CS/Le NPs, entrapment efficiency (EE) and drug loading percentage (DL%)

Briefly, Amicon Ultra-15 filters (30 kDa, Millipore^®^) were used to filter the suspension of M-CS/Le NPs. The non-entrapped melatonin was quantified in the collected filtrate (279 nm) according to Lopes Rocha Correa et al. (2020). By determining the quantity of melatonin that was not encapsulated in the nanoparticles, the EE was indirectly determined. To do this, the produced nanoparticle solution was filtered, then it was centrifuged for 30 min at 2000 rpm and 4 °C. The EE% = [(Whole melatonin–Unloaded melatonin)/Total melatonin] × 100. The formula used to measure DL% = [(Whole melatonin–Unloaded melatonin)/quantity of produced nanoparticles] × 100. The melatonin release (%) from M-CS/Le NPs was measured using the sink conditions, which takes into account melatonin’s solubility in water (Hafner et al. 2011). M-CS/Le NPs were suspended in acetate buffer (90 µg/ml). Melatonin released was followed up to 48 h. The amount of melatonin was calculated using absorbance at 279 nm. Acetate buffer was chosen for measuring the melatonin release from M-CS/Le NPs. Melatonin is a highly lipophilic molecule with limited water solubility. This low solubility in aqueous solutions can lead to its precipitation and inaccurate release measurements. Acetate buffer helps maintain consistent solubility and prevents its aggregation, ensuring accurate release kinetics.

### Examination of the safety of M, CS/Le NPs and M-CS/Le NPs in vitro

#### The antioxidant activity

It was assessed by four methods to determine the scavenging effect.

- For 1, 1-diphenyl-2-picryl hydrazyl (DPPH) method: ascorbic acid served as a standard to measure the antioxidant activity of the generated M-CS/Le NPs. In summary, a DPPH-methanol solution (1 milliliter/0.1 mm) was combined with different concentrations of the sample. After shaking, the solutions were kept at 25 °C for 30 min. The DPPH scavenging activity % = [(Ab1-Ab2)/Ab1]X100, where, the absorbance (Ab) was determined at 517 nm. The absorbance of the control and sample was Ab1 and Ab2, respectively.
- For measuring the hydrogen peroxide (H_2_O_2_) scavenging effect: 600 µL of H_2_O_2_ (2 mM) and 300 µL of saline solution (pH = 7.4) were mixed with 100 µL of the sample at different concentrations followed by shaking and the absorbance was detected at 230 nm. Ascorbic acid was utilized as the standard and saline as the control. The scavenging % = [(Ab1-Ab2)/Ab1]X100. Ab2 was the sample absorbance while Ab1 was the control absorbance.
- For measuring the hydroxyl radical (OH^-^) scavenging effect: 100 µL of the sample at varying concentrations (in methyl alcohol) were mixed with saline buffer (450 µL), deoxyribose (150 µL), FeSO_4_/EDTA (150 µL), H_2_O_2_ (150 µL), and deionized H_2_O (525 µL). Equal volume (750 µL) of trichloroacetic acid and thiobarbituric acid (2.8% and 1%, respectively) were added to terminate the reaction after four hours. After ten minutes of heating the combination, it was left to cool, and the result was determined at 532 nm. Ascorbic acid served as the standard and methanol as the control. The scavenging %= [(Ab1-Ab2)/Ab1]X100. Ab1 and Ab2 were the absorbance of the control and sample, respectively.
- For measuring the superoxide radical (O_2_^−^) scavenging effect: 100 µL of the sample at varying concentrations (in methyl alcohol) were mixed with 1000 µL Tris–HCl (16 mM, pH = 8), 100 µL nitro blue tetrazolium (50 µM), 1000 µL nicotinamide adenine dinucleotide (78 µM), and 1000 µL phenazinemethosulphate (10 µM). After 5 min, the mixture’s absorbance at 560 nm was determined. The formula [(Ab1-Ab2)/Ab1]X100 was utilized to calculate the inhibition of O_2_^−^ generation. Ab1 and Ab2 were the absorbance of the control and sample, respectively.

#### The anti-inflammatory (membrane stabilization) activity

Erythrocytes suspension was prepared from fresh blood (drown from rat with heparin vaccum tube). Samples were mixed with 5 mL distilled H_2_O (hypotonic solution) or 5 mL of saline (isotonic solution) with different doses. The different mixtures were allowed to incubate for 60 min followed by the centrifugation at 2000 rpm for 5 min. The supernatant was used to evaluate the amount of released hemoglobin (at 560 nm). 5 mL of indomethacin (200 µg/ml) was used as a standard. The hemolysis inhibition %= 1 − [(Ab2– Ab1)/(Ab3– Ab1)] × 100. Ab1 and Ab2 were the sample’s absorbance in the isotonic and the hypotonic solution, respectively; where Ab3 was the control absorbance.

#### The anti-microbial activity

The antimicrobial activity of the sample was evaluated through the disc inhibition zone, in which 100 µL of 10^6^ CFU bacterial suspensions were added to 25 mL of agar plates. A hole (6 mm), in the plates, was created by the aid of a sterile tip. One hundred µL of the sample (10 mg/ml) was added to the holes followed by incubation then measuring the inhibition zone diameter (mm). The pathogens were obtained from the Microbiological Resources Center (Cairo MIRCEN) [*Staphylococcus aureus* (ATCC 6538), *Bacillus subtilis* (ATCC 6633), *Candida albicans* (ATCC 10,221), *Pseudomonas aeruginosa* (ATCC 90,274), *Escherichia coli* (ATCC 8739) and *Mucor*].

#### The cytotoxicity activity

The effect of produced M-CS/Le NPs on the viability of cells was evaluated by the MTT [3-(4,5-dimethylthiazol-2-yl)-2-5-diphenyltetrazolium bromide] method. Rat Epidermal Keratinocytes (R2100, ScienCell, USA) were cultures at 37 ^o^C and 5% CO_2_ for 24 h for the cells monolayer’s development. The culture media was supplemented with varying concentration of the samples followed by two hours of incubation. The MTT (5 mg/ml) was added to the cultures for dissolving formazan that was evaluated at 560 nm.

### Examination of the M-CS/Le NPs wound healing activity in vivo

#### Induction of diabetes

Prior to administering STZ, the animals were fasted for six hours. STZ (32.5 mg/ml) solution was prepared by dissolving in 50 mM Na_3_C_6_H_5_O_7_·2H_2_O (pH = 4.5). An intravenous dosage of 65 mg/kg (2.0 ml/kg) of STZ solution was given to the rats. Rats were fed 10% sucrose water on the first day, only, following the treatment with STZ. The animals were fasted for six hours on the tenth day, and blood samples (from the tail’s vein) were used in measuring the fasting blood glucose (FBG). Animals were classified as diabetic when the FBG level was higher than 160 mg/dl relative to that of the control group. Serum fructosamine (FTA) was tested using a Rat FTA ELISA Kit (MBS2601586, MyBioSource, USA) to ascertain the average blood sugar level throughout the preceding three weeks.

#### Excision wound model

The animals were put under anesthesia through the intraperitoneal injection with ketamine and xylazine (30 and 10 mg/kg, respectively); their backs were shaved and cleaned with ethyl alcohol (70%). After designating a region on the rat’s back, a full thickness skin wound of about 2 cm (diameter) was made. An inoculation of 10 µl of *Staphylococcus aureus* (10^9^ CFU) was used to cause infections in wounds (Rajoo et al. 2021).

#### Experimental design

**Fig. 1:**
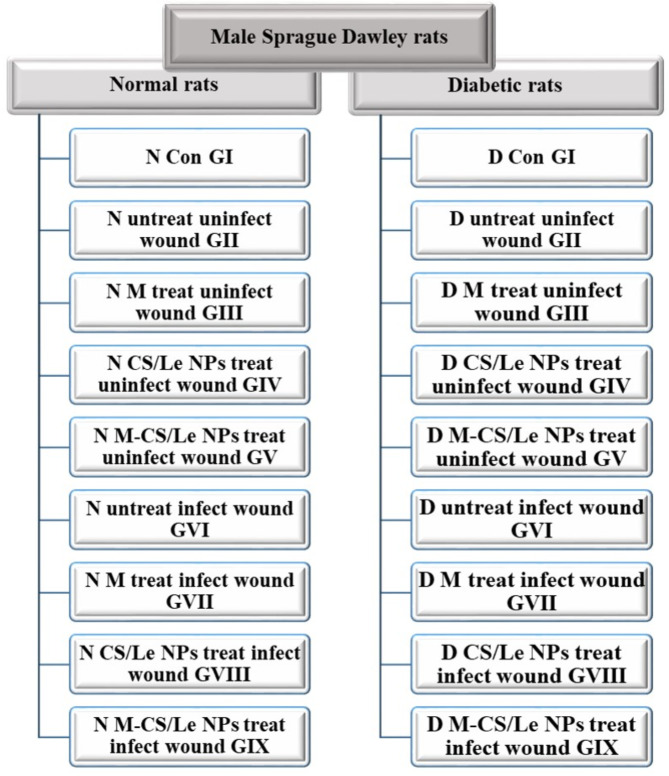
experimental groups’ design, animals were divided into normal and diabetic rats. Each division was divided into nine groups (9 rats/group)

Ten weeks old and weighing between 180 and 200 g, male Sprague Dawley rats were acquired from the National Organization for Drug Control and Research (NODCAR, Cairo, Egypt). The research and experimental procedures on animals was conducted adhering to the ARRIVE standards. The animals were separated into two primary categories: the normal (NG) and the diabetic (DG) animals. Additionally, each category included nine groups, each with nine rats (Fig. 1):

- Group I: Control rats (Con GI)
- Group II: Rats with untreated uninfected wound (untreat uninfect wound GII)
- Group III: Rats with uninfected wound treated with melatonin (10 mg/kg) (M treat uninfect wound GIII)
- Group IV: Rats with uninfected wound treated with unloaded chitosan/lecithin nanoparticles (5 mg/kg) (CS/Le NPs treat uninfect wound GIV)
- Group V: Rats with uninfected wound treated with melatonin loaded-chitosan/lecithin nanoparticles (5 mg/kg) (M-CS/Le NPs treat uninfect wound GV)
- Group VI: Rats with infected untreated wound (untreat infect wound GVI)
- Group VII: Rats with infected wound treated with melatonin (10 mg/kg) (M treat infect wound GVII)
- Group VIII: Rats with infected wound treated with unloaded chitosan/lecithin nanoparticles (5 mg/kg) (CS/Le NPs treat infect wound GVIII)
- Group IX: Rats with infected wound treated with melatonin loaded-chitosan/lecithin nanoparticles (5 mg/kg) (M-CS/Le NPs treat infect wound GIX).

After wound induction, animals received topical administration of free melatonin, CS/Le NPs and M-CS/Le NPs twice a day (one every 12 h) for 14 successive days. The wound area percent (%) was calculated on the 1st, 7th and 14th days after wound induction. From each group, three animals were anesthetized with sodium pentobarbital (50 mg/kg) on the 1st, 7th and 14th days after wound induction in order to collect skin samples for measurements of oxidative stress and immunological parameters.

### Calculation of wound area percentage

The wound area % was calculated until the point of closure. Using transparent graph paper and a marker, the continuous reduction in the wound area was followed up on the 1st, 7th and 14th days after wound induction. The following formula was used to get the wound area %:

$$ $$$${\text{Wound}}\;{\text{area}}\;\% {\text{ = }}\frac{{{\text{area}}\:{\text{of}}\:{\text{wound}}\:{\text{on}}\:\left( {\text{n}} \right)\:{\text{day}}}}{{{\text{area}}\:{\text{of}}\:{\text{wound}}\:{\text{on}}\:{\text{zero}}\:{\text{day}}}}{\text{X}}\;{\text{100}}$$$$ $$

### Skin homogenate preparation

Half g of skin sample was homogenized with saline solution (1 mL) and subjected to 2 freeze/thaw cycles followed by centrifugation for 10 min at 2000 rpm. The supernatant was used to evaluate the different biochemical parameters in skin tissue homogenates.

### Immunological measurements

IL-1β and TNF-α levels (in skin homogenates) were determined by rat ELISA kits (ab100768, Abcam, USA and CSB-E11987r, Cusabio, USA; respectively) to evaluate the immunological impact of M-CS/Le NPs on wound healing in various experimental groups.

### Oxidative stress measurements

To examine the antioxidant capability of M-CS/Le NPs in vivo, rat ELISA Kits (E-EL-R1424 and E-EL-0060, respectively; Elabscience, USA) were used to quantify the superoxide dismutase (SOD) and the lipid peroxidation marker (malondialdehyde, MDA) levels in skin homogenates.

### Effect on apoptotic proteins

The effect of M-CS/Le NPs on the levels of intracellular apoptotic proteins was measured in skin tissue homogenates. Rat ELISA kits was used in the measurement of the levels of the pro-apoptotic proteins (Bax, p53, caspase 3, and caspase 8) and the anti-apopttotic protein Bcl2 (MBS935667, MBS723886, MBS018987, MBS260539 and MBS2515143 MyBioSource, USA; respectively)].

### Statistical analysis

ANOVA (one-way analysis of variance) was employed to examine how the nanoparticles affected the markers under investigation. Bonferroni’s post-hoc test was used to find the differences between means (*P* < 0.05).

## Results

### Characterization of NPs

**Fig. 2:**
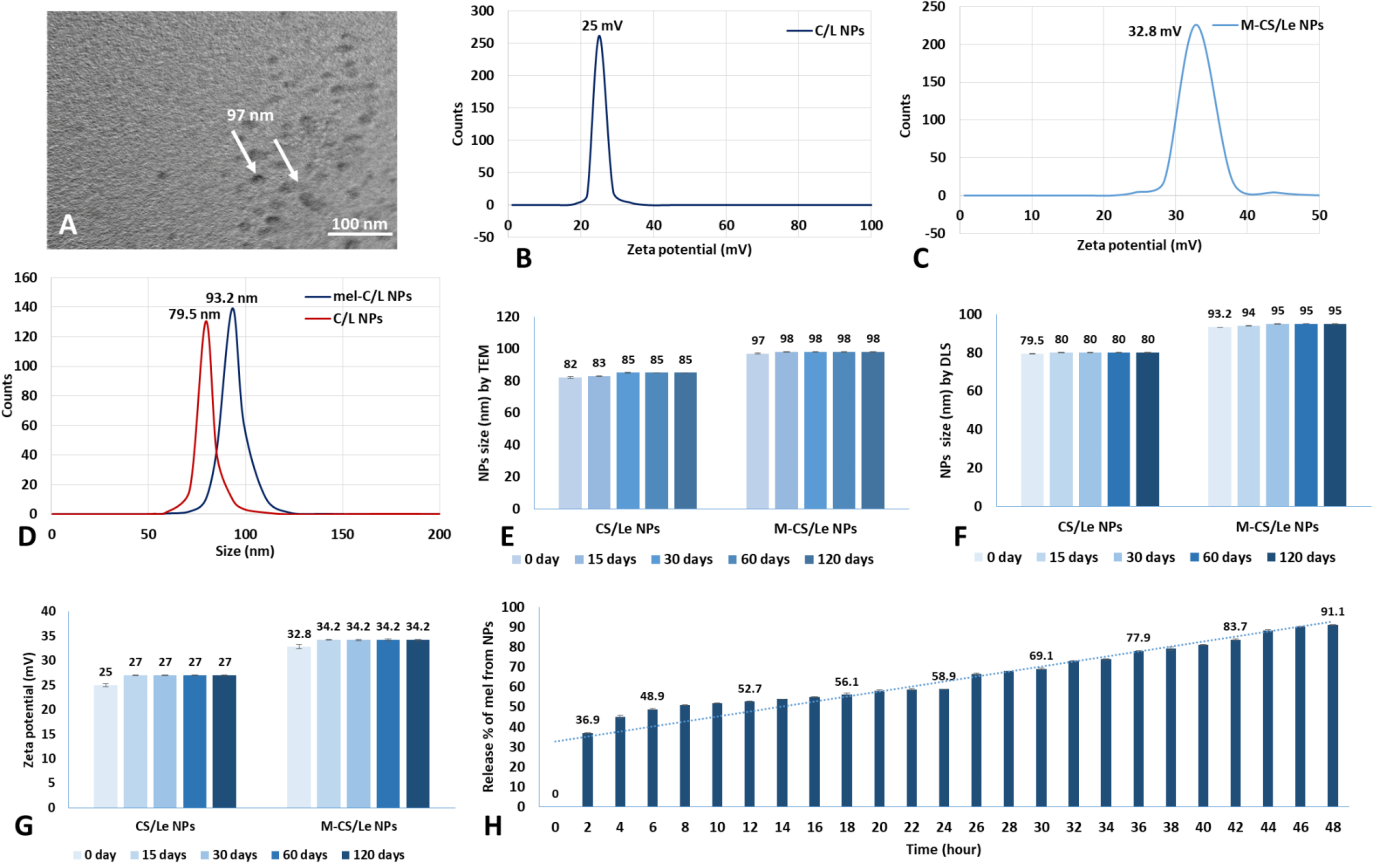
physical characterization showing size (nm) of M-CS/Le NPs (**A**) under TEM on 0 day after synthesis; zeta potential of CS/Le NPs (**B**) and M-CS/Le NPs (**C**) on 0 day after synthesis; hydrodynamic size of CS/Le NPs and M-CS/Le NPs (**D**) on 0 day after synthesis; NPs size (nm) by TEM (**E**), NPs size (nm) by DLS (**F**) and zeta potential (mV) (**G**) on the 0, 15, 30, 60 and 120 days after synthesis; release % of melatonin from M-CS/Le NPs (**H**)

TEM image showed that M-CS/Le NPs has a size of 97 nm after melatonin loading (on 0 day after synthesis) (Fig. 2A). The hydrodynamic size of NPs (79.5 and 93.2 nm for CS/Le NPs and M-CS/Le NPs, respectively), obtained by DLS (Fig. 2D), was similar to that obtained from TEM (97 nm for M-CS/Le NPs). The follow up of size and zeta potential (up to 120 days) revealed the nanoparticles stability (Fig. 2E, F and G). The size of NPs started to slightly increase after fifteen days of preparation; then, stayed constant for 120 days post preparation. On the 0 day after synthesis, CS/Le NPs has a positive zeta potential (25 mV) (Fig. 2B) that increased to 32.8 mV after melatonin loading (Fig. 2C). The zeta potential (surface charge) of CS/Le NPs increased to 27 mV, on the 15 days post synthesis, then stayed constant up to 120 days. On the other hand, the zeta potential of M-CS/Le NPs increased to 34.2 mV and stayed constant up to 120 days (Fig. 2G). The release % of melatonin from M-CS/Le NPs (Fig. 2H) showed a controlled and sustainable release up to 48 h (91.1%).

#### The in vitro characterization of NPs

**Fig. 3:**
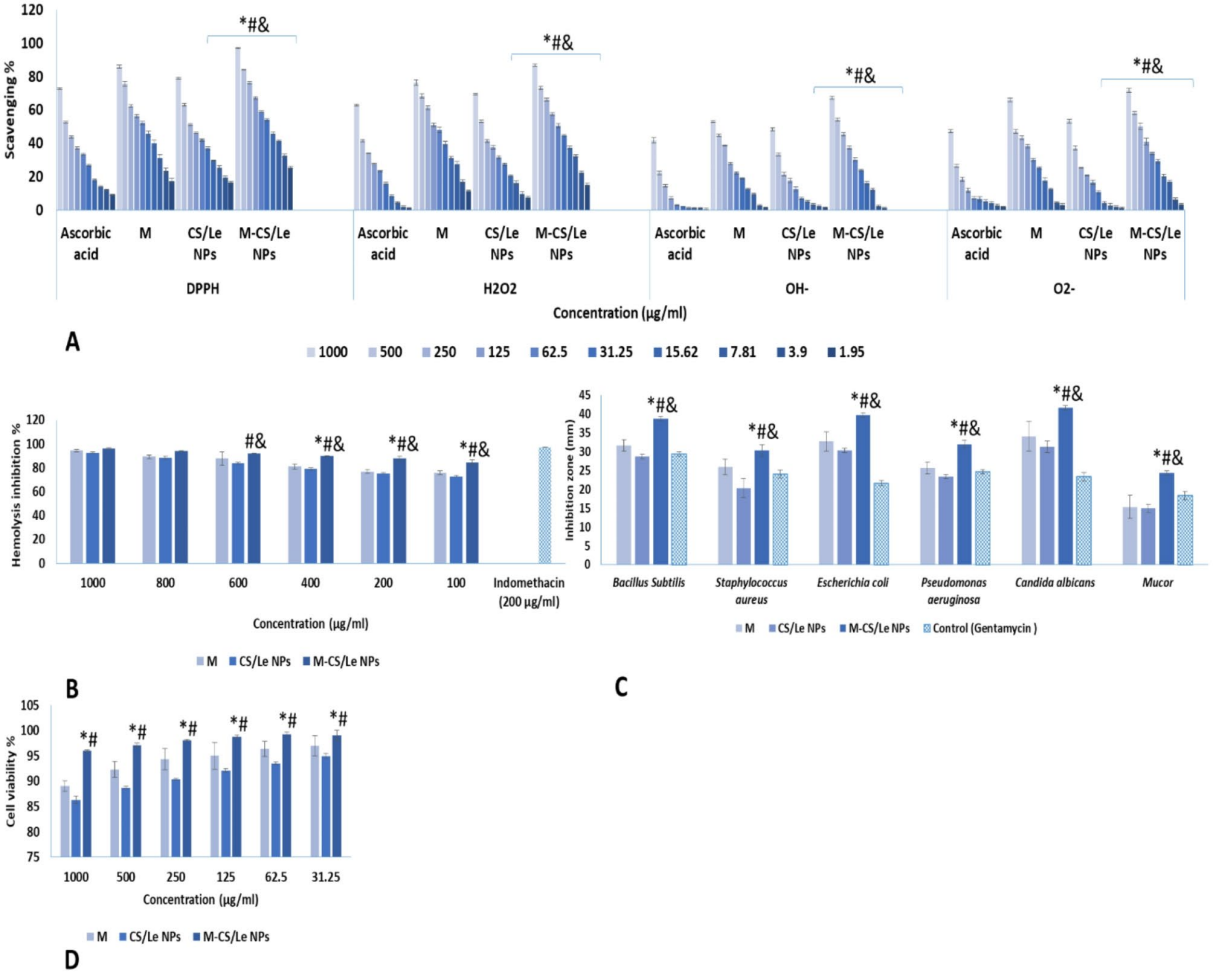
in vitro characterization of M, CS/Le NPs and M-CS/Le NPs showing the antioxidant activity (**A**) where *, # and & represented significance with respect to ascorbic acid, M and CS/Le NPs, respectively (*p* < 0.05); the anti-inflammatory activity (**B**) where *, # and & represented significance with respect to indomethacin, M and CS/Le NPs, respectively (*p* < 0.05); the microbial activity (**C**) where *, # and & represented significance with respect to gentamycin, M and CS/Le NPs, respectively (*p* < 0.05) and the cell viability activity (**D**) where * and # represented significance with respect to M and CS/Le NPs, respectively (*p* < 0.05). Results were represented as mean ± SD

M-CS/Le NPs showed a powerful antioxidant activity; where, it succeeded in scavenging DPPH, H_2_O_2_, OH^−^ and O^2−^ in a dose proportional way. The antioxidant capabilities of M-CS/Le NPs were significantly elevated than those of ascorbic acid, M and CS/Le NPs (Fig. 3A). The prepared M-CS/Le NPs significantly prevented the RBCs’ hemolysis in a dose-proportional manner in comparison to indomethacin. The hemolysis inhibition % of M-CS/Le NPs was significantly higher than those of M and C/L NPs at 100, 200 and 400 µg/mL (Fig. 3B). A marked anti-microbial activity was noticed for M-CS/Le NPs against all tested bacterial and fungal species. The inhibition zones were bigger with M-CS/Le NPs than those with gentamycin; and also, bigger than those with CS/Le NPs and M (Fig. 3C). In the MTT assay, the M-CS/Le NPs preserved the cells and showed a significant high cell viability % (Fig. 3D).

### Blood glucose and fructosamine

**Fig. 4:**
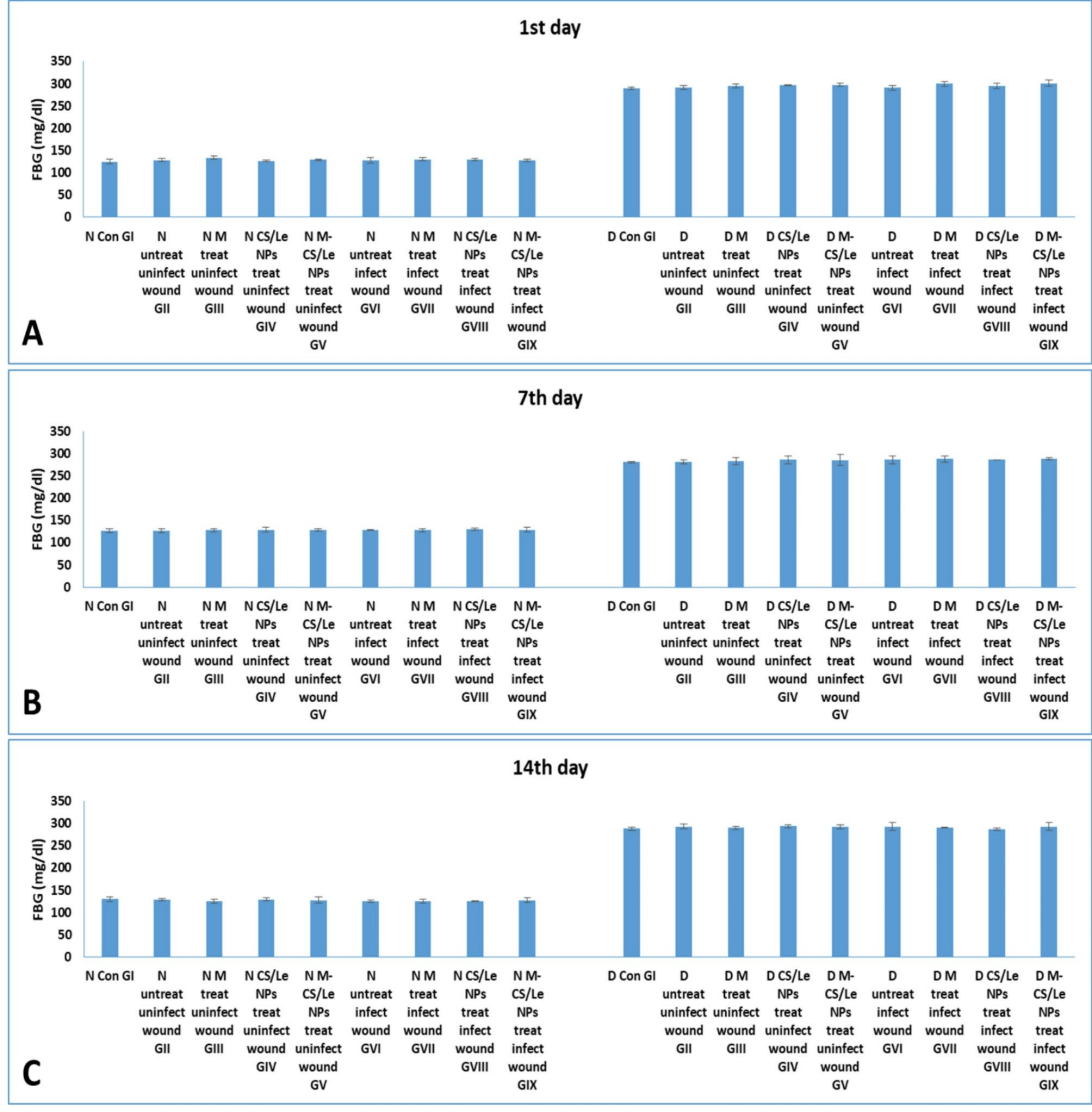
levels of FBG in normal and diabetic groups on the 1st (**A**), 7th (**B**) and 14th (**C**) days. Data were expressed as mean ± SD

**Fig. 5:**
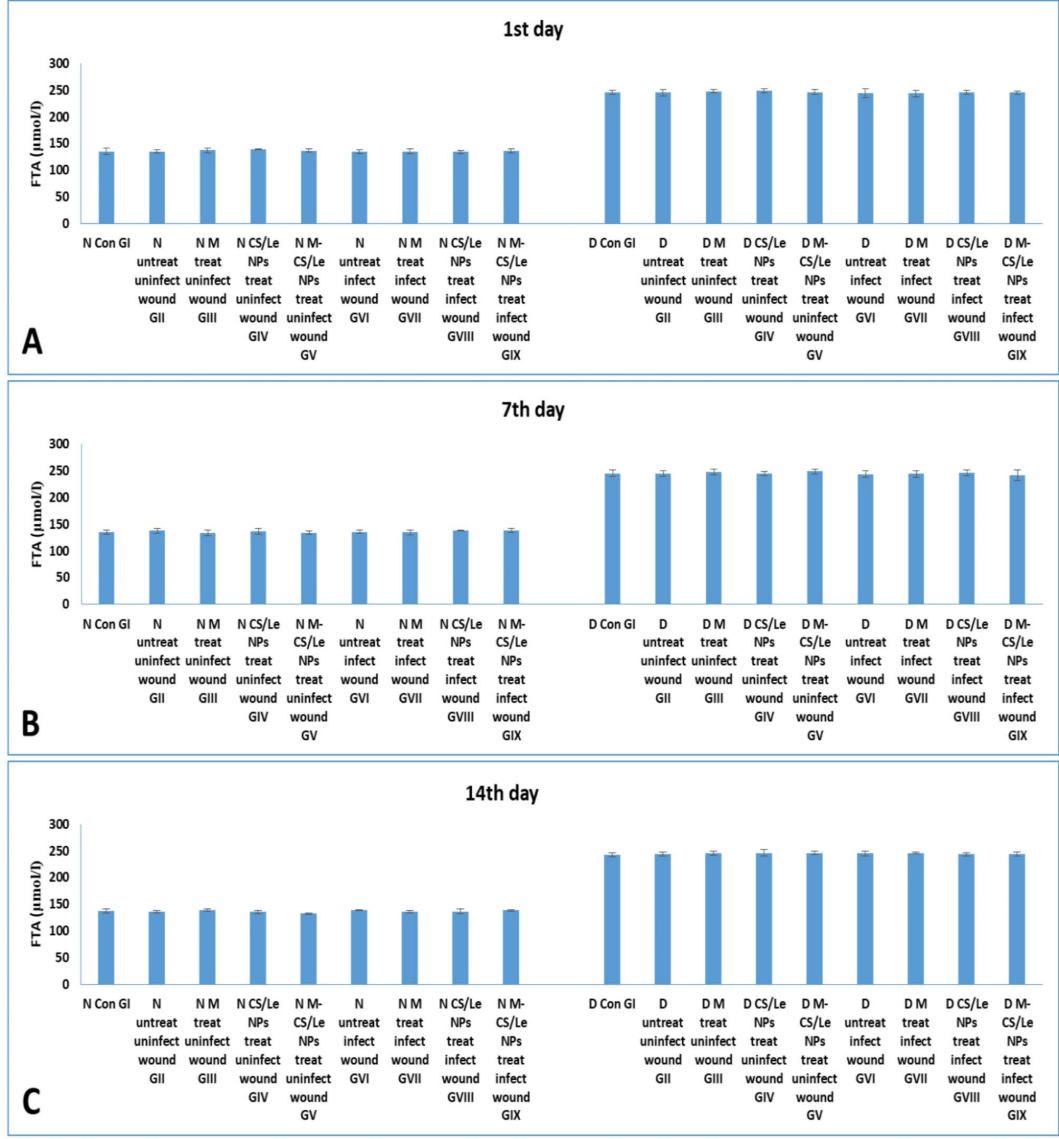
levels of FTA in normal and diabetic groups on the 1st (**A**), 7th (**B**) and 14th (**C**) days. Data were expressed as mean ± SD

Diabetes was confirmed by the measurements of both FBG and FTA (which indicated level of blood glucose in the previous three weeks). At all experimental periods, no significance difference was detected in the FBG or FTA levels among normal non-diabetic groups (GI to GIX). A high increase in FBG and FTA levels was observed in diabetic groups (GI to GIX) on the 1st, 7th and 14th day PI (Figs. 4 and 5) when compared to non-diabetic groups.

### Oxidative stress

**Fig. 6:**
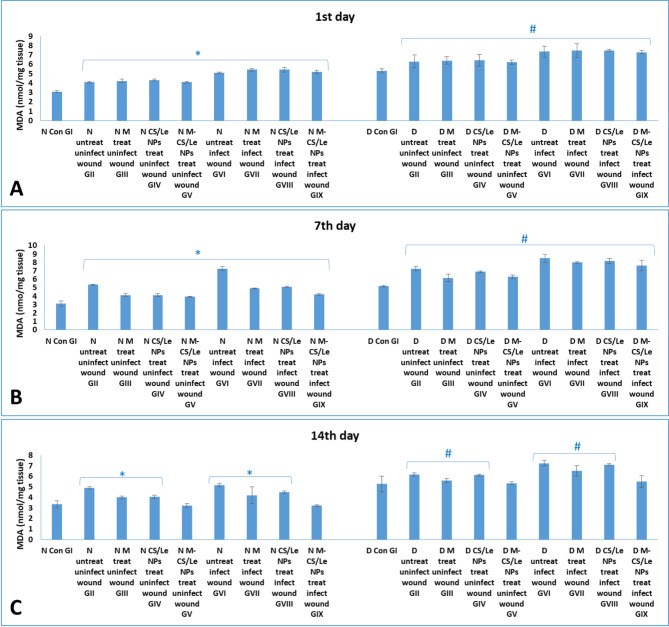
levels of MDA in normal and diabetic groups on the 1st (**A**), 7th (**B**) and 14th (**C**) days. Data were expressed as mean ± SD, where in normal groups, * represented significance (*P* < 0.05) with respect to normal control group I; and in diabetic groups, # represented significance (*P* < 0.05) with respect to diabetic control group I

**Fig. 7:**
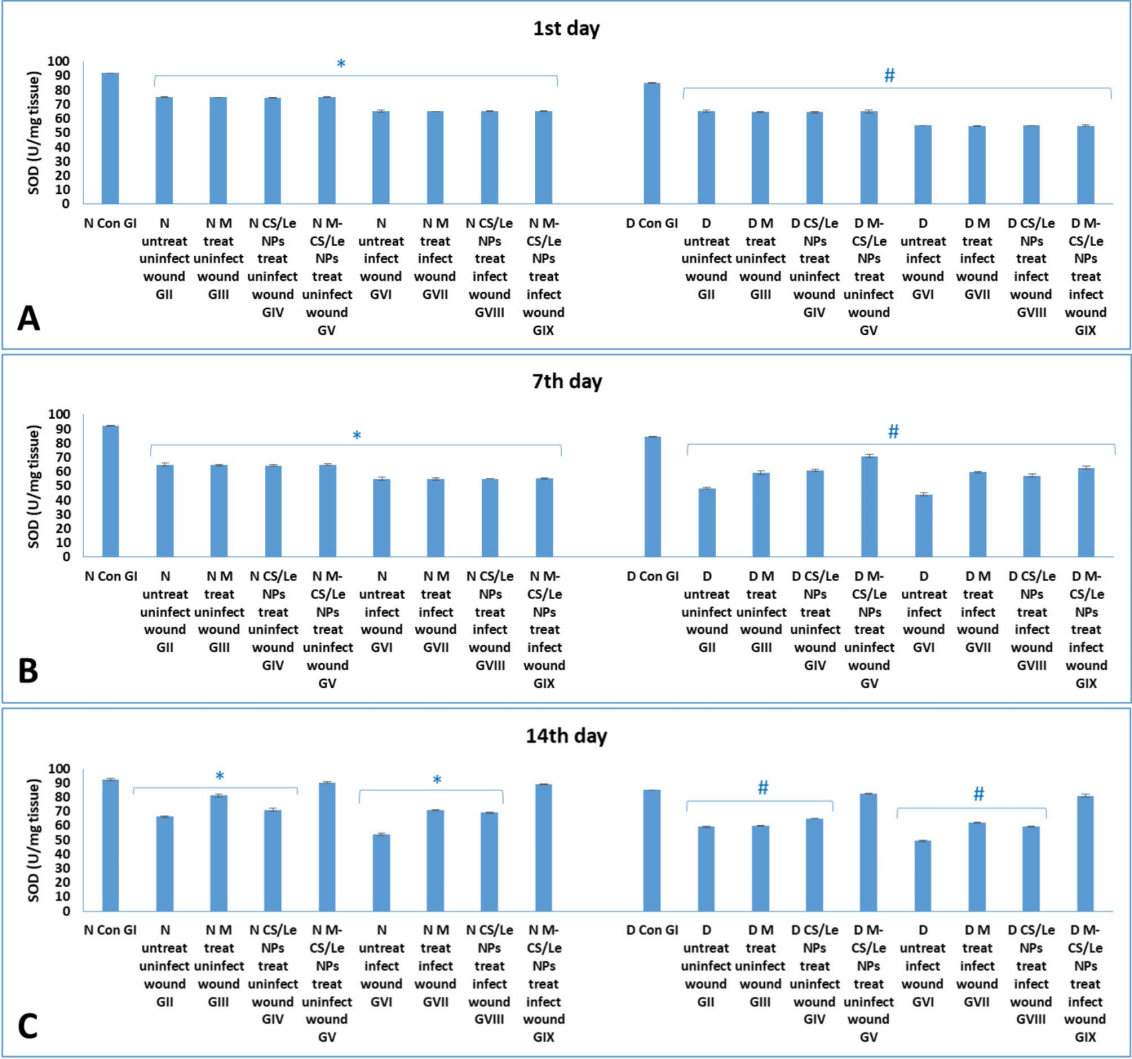
levels of SOD in normal and diabetic groups on the 1st (**A**), 7th (**B**) and 14th (**C**) days. Data were expressed as mean ± SD, where in normal groups, * represented significance (*P* < 0.05) with respect to normal control group I; and in diabetic groups, # represented significance (*P* < 0.05) with respect to diabetic control group I

**On the 1st day**: in normal non-diabetic groups, the induction of wounds significantly elevated the MDA level and significantly reduced the SOD levels. This elevation in the oxidative stress and the disturbance in the antioxidant enzyme levels were significantly magnified with wound infection. It was obvious that diabetes induction significantly elevated MDA levels in diabetic groups than in normal non-diabetic ones. **On the 7th day PT**: the rise in the level of MDA and the decrease in the level of SOD reached the highest peak among all experimental periods. The highest MDA level and lowest SOD level were observed in diabetic GVI with non-treated infected wound. **On the 14th day PT**: treatment of wounds with free melatonin or CS/Le NPs, alone, did not succeed in normalized the MDA and SOD levels to be similar to those of normal control GI. M-CS/Le NPs administration in treated non-diabetic or diabetic groups (GV and GIX) successfully returned the MDA and SOD levels to be like to those of their corresponding control groups (Figs.  6 and 7).

### Pro-inflammatory cytokines

**Fig. 8:**
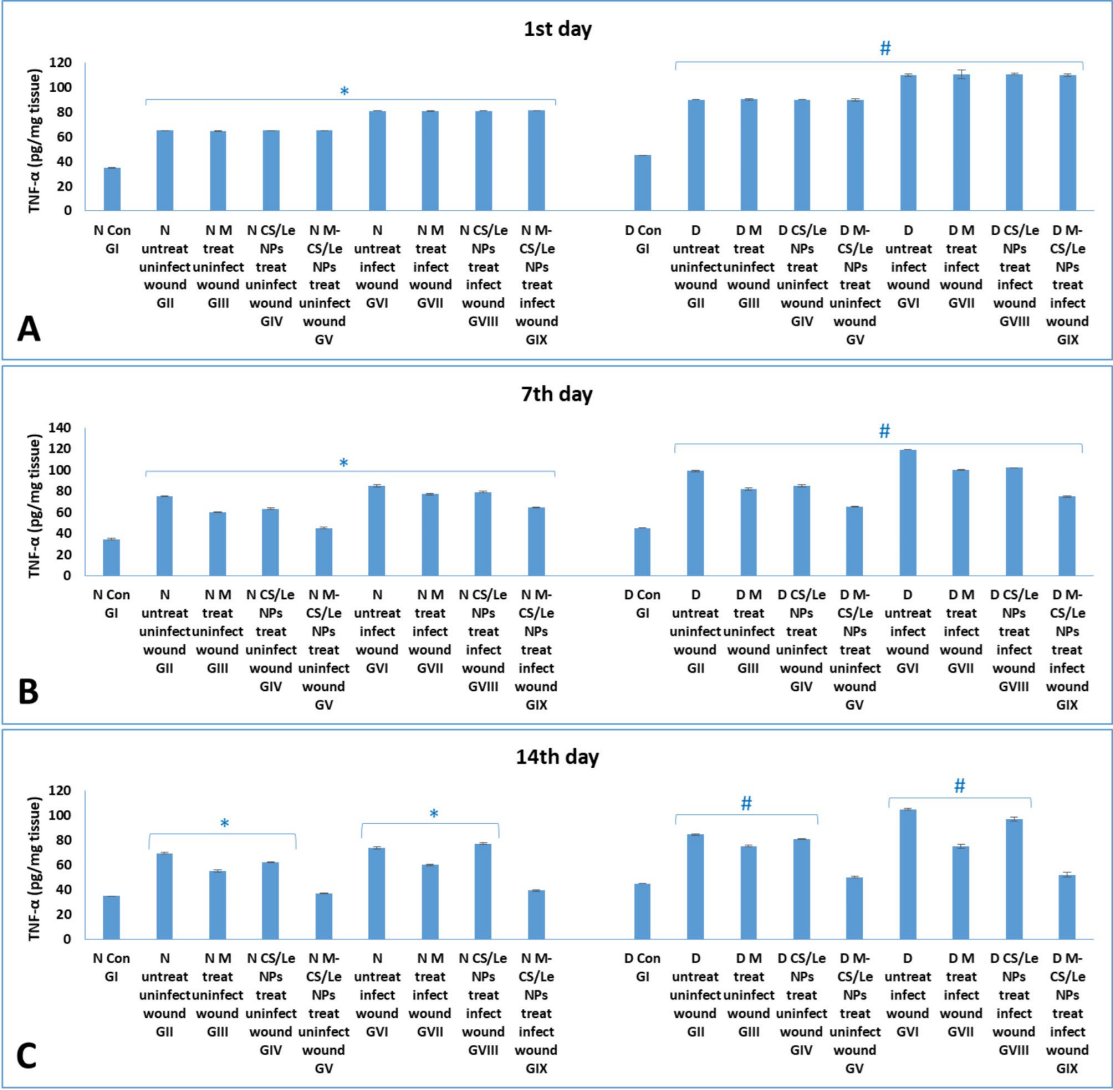
levels of TNF-α in normal and diabetic groups on the 1st (**A**), 7th (**B**) and 14th (**C**) days. Data were expressed as mean ± SD. In normal groups, * represented significance (*P* < 0.05) with respect to normal control group I; and in diabetic groups, # represented significance (*P* < 0.05) with respect to diabetic control group I

**Fig. 9:**
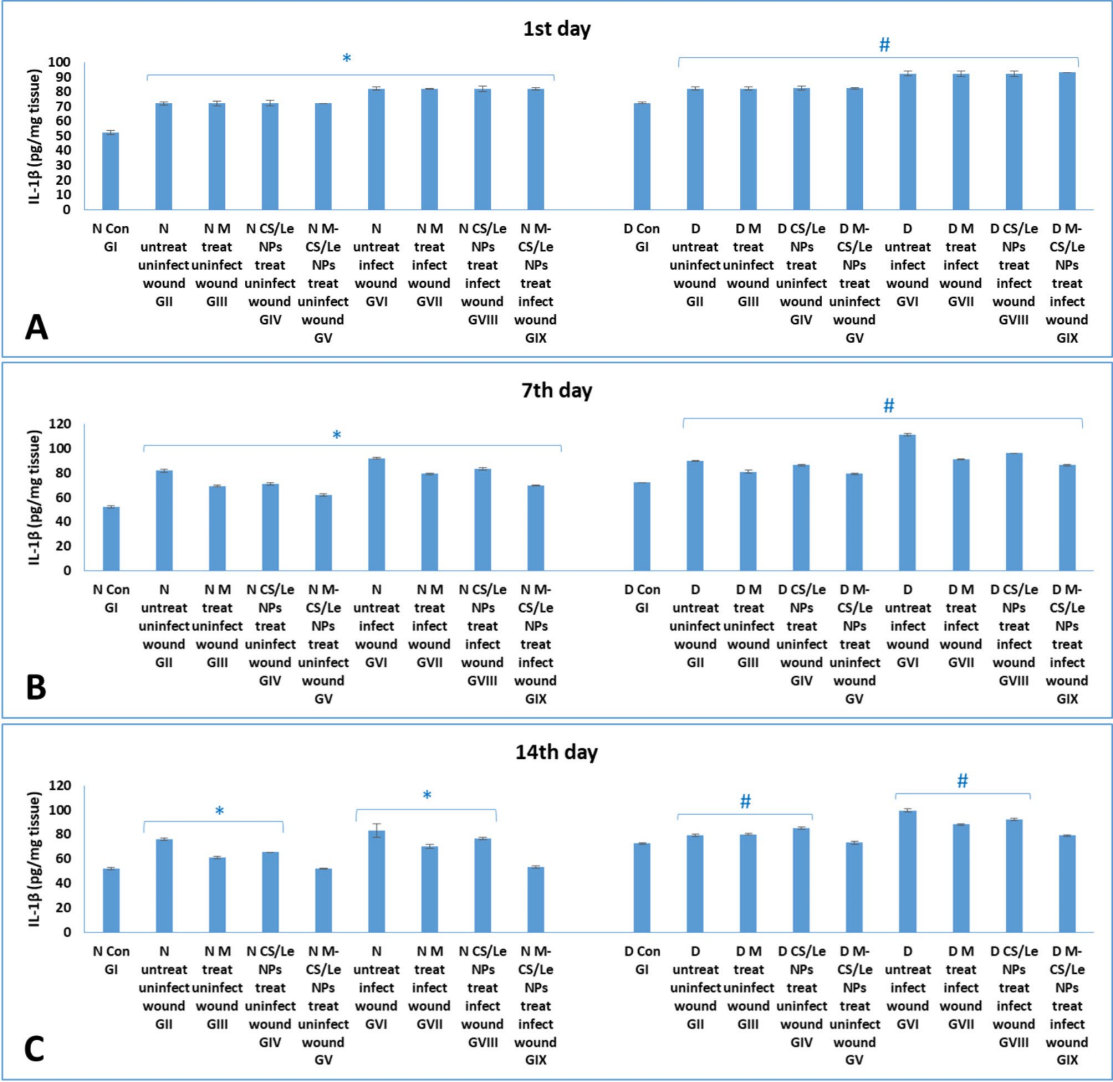
levels of IL-1β in normal and diabetic groups on the 1st (**A**), 7th (**B**) and 14th (**C**) days. Data were expressed as mean ± SD. In normal groups, * represented significance (*P* < 0.05) with respect to normal control group I; and in diabetic groups, # represented significance (*P* < 0.05) with respect to diabetic control group I

**On the 1st day**: high levels of the inflammatory cytokines (TNF-α and IL-1β) were observed in skin tissue homogenates of diabetic groups than of non-diabetic groups. Groups with infected wounds showed higher cytokine levels than those of uninfected wounds. **On the 7th day PT**: in all groups, the highest cytokines levels were observed on this time. Moreover, diabetic GVI (with untreated infected wounds) showed the highest cytokines levels among all experimental groups. **On the 14th day PT**: level of cytokines in non-diabetic and diabetic groups treated with M-CS/Le NPs (GV and GIX) were similar to those of their corresponding control GI (Figs.  8 and 9).

### Apoptosis measurements

**Fig. 10:**
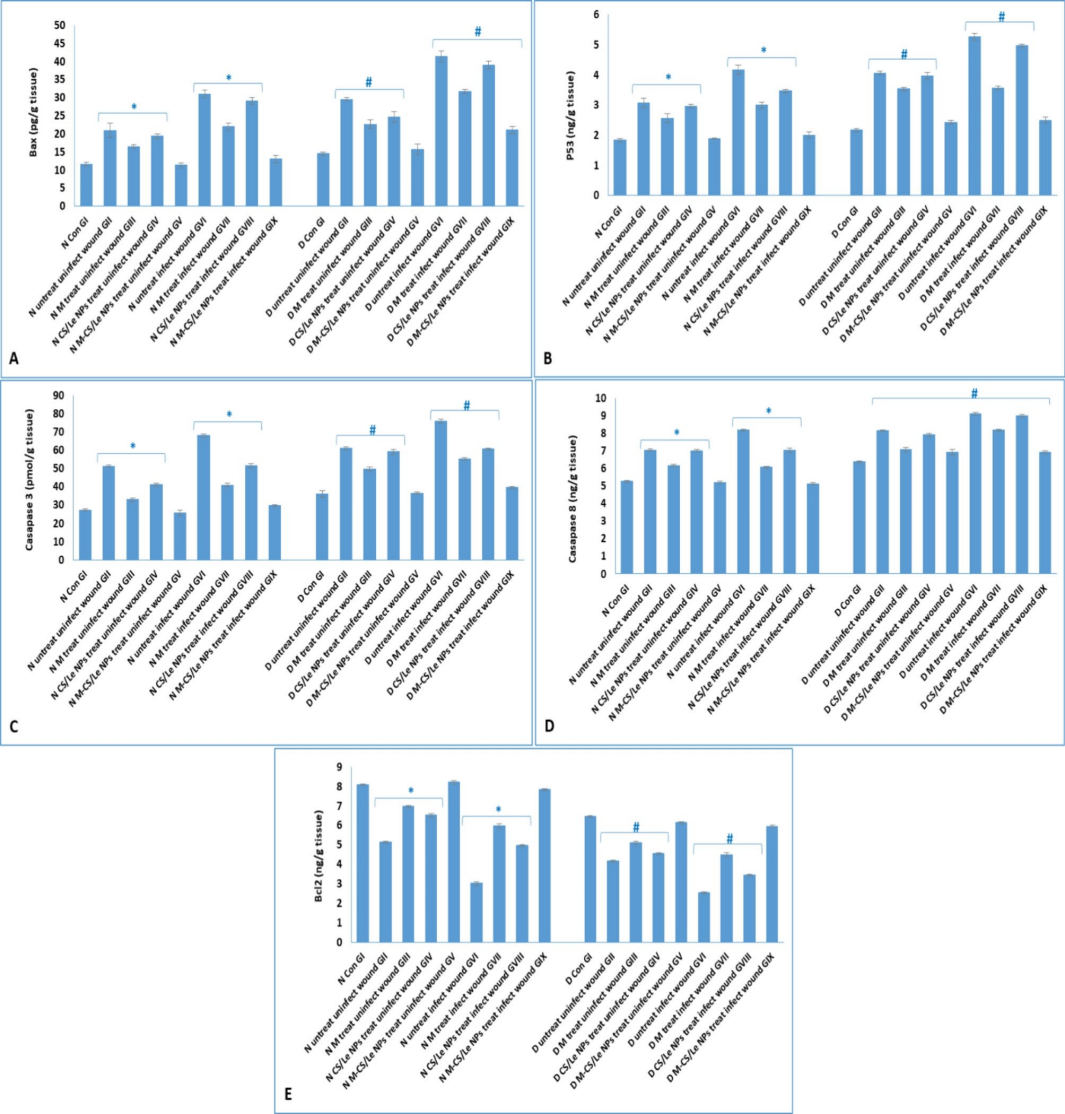
levels of intracellular apoptotic proteins [Bax (**A**), P53 (**B**), casapase 3 (**C**) and casapase 8 (**D**)] and anti-apoptotic intracellular protein [Bcl2 (**E**)] in skin tissue. Data were expressed as mean ± SD. In normal groups, * represented significance (*P* < 0.05) with respect to normal control group I; and in diabetic groups, # represented significance (*P* < 0.05) with respect to diabetic control group I

Wound induction, in non-diabetic untreated uninfected control GII, significantly raised the levels of the pro-apoptotic proteins (Bax, p53, casapase3 and 8); and significantly decreased the anti-apoptotic protein Bcl2 level in skin tissue homogenates. The previous disturbance (that resulted from wound induction) was significantly elevated in untreated non-diabetic and diabetic infected groups (N GVI and D GVI). Although the administration of free melatonin or CS/Le NPs slightly ameliorated the levels of apoptotic markers in skin tissue, their effects were not effective when compared to that of M-CS/Le NPs. Administration of M-CS/Le NPs was significantly effective in decreasing the intracellular apoptotic proteins levels and increasing the anti-apoptotic protein Bcl2 level in treated diabetic and non-diabetic groups (Fig. 10).

### Wound area %

**Fig. 11:**
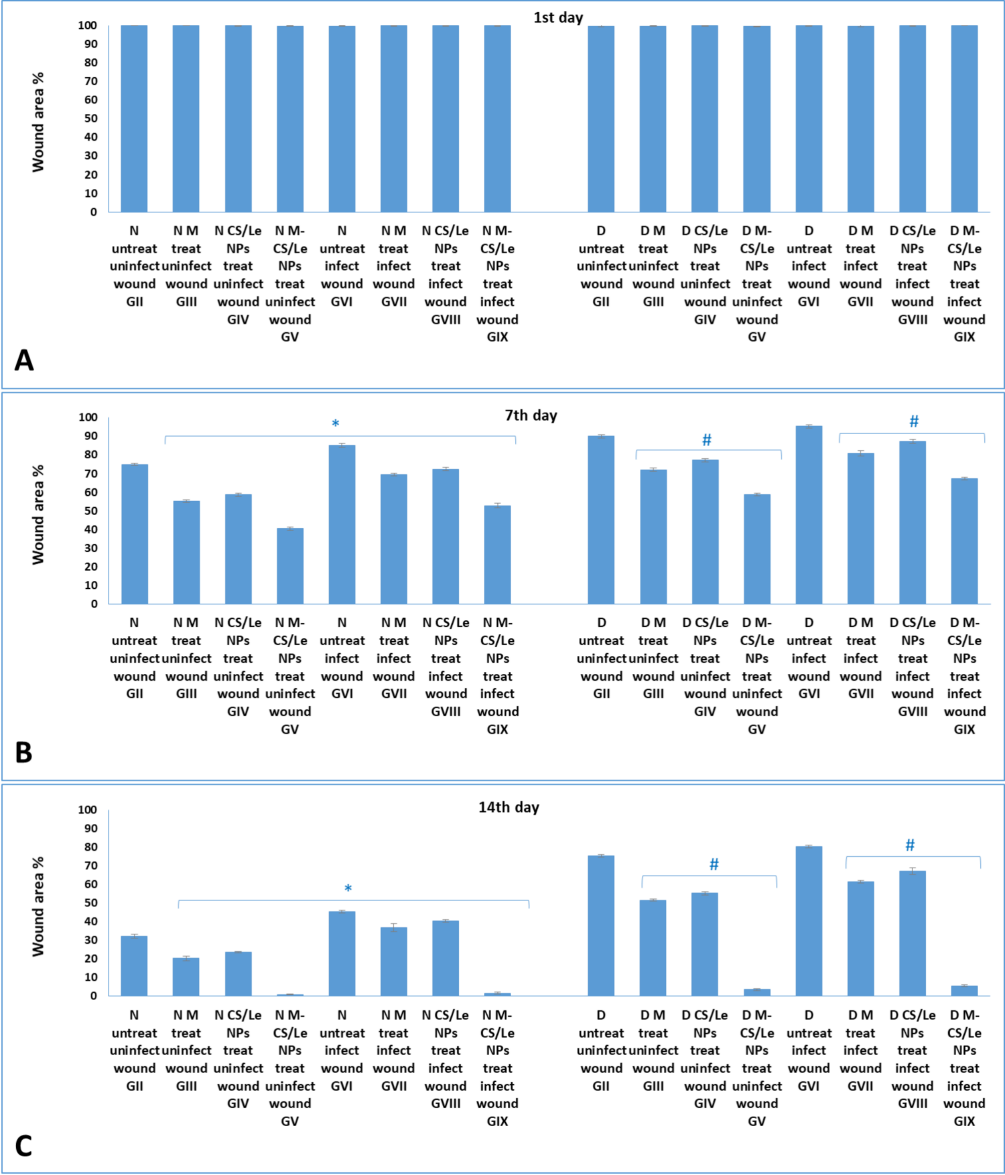
wound area % in different experimental groups on the 1st day (**A**), 7th day PT (**B**) and 14th day PT (**C**). Data were expressed as mean ± SD. In normal groups, * represented significance (*P* < 0.05) with respect to normal control group I; and in diabetic groups, # represented significance (*P* < 0.05) with respect to diabetic control group I

According to Fig. 11, the wound area % has been linked to both type (free melatonin, CS/Le NPs or M-CS/Le NPs) and time duration (days) of treatment. Upon wound induction (1st day), no significant difference was noticed in the wound area % among all tested groups. On the 7th day PT, a significant decrease in the wound area % was noticed in all groups when compared to that of the 1st day. On the other hand, M-CS/Le NPs successfully aided in the wound healing process and decreased the wound area % in healthy and diabetic treated groups more than free melatonin or CS/Le NPs.

## Discussion

Diabetic individuals suffer from non-healing wounds with severe inflammatory response and retarded epithelialization. Patients with injuries in blood vessels and nerves are more prone to develop foot issues, where sensory loss is common. Small sores on the foot might form and go undetected. Later, they grow into deeper ulcers that take longer time to heal, and problems such as infection occur, necessitating amputation owing to infection spreading to the underlying tissue and bone. Foot ulcers affect 15 to 25% of individuals (Singh et al. 2005; Pendsey 2010), with 6% requiring hospitalisation to cure (Carrington et al. 2001).

The skin serves as a shield against the surroundings, and removing any part of it can result in impairment. The healing process is a multi-phased and complicated dynamic process. The healing process is linked to a number of local and systemic factors, comprising the age, the used treatment, patient’s nutrition state, oxygen level and the used dressing. One of medicine’s most significant aims is to have a rapid recovery with minimal scarring. Furthermore, reducing the recovery time is essential since it minimizes the risk of infection as well as complications and expenses. Therefore, the present study aimed to prepare M-CS/Le NPs and test its wound healing activity in full thickness induced-wound diabetic animal model (male Sprague Dawley rats). The wounds were challenged with *Staphylococcus aureus* infection to examine the antibacterial effect of M-CS/Le NPs.

The CS/Le ratio, in the present work, was 1:1, and the procedure followed for M-CS/Le NPs preparation was in line with Lopes Rocha Correa et al. (2020). Dynamic light scattering (DLS) was used to measure the size of the nanoparticles. The particle size were measured using a transmission electron microscope (TEM). The physical characterization (size and surface charge) was performed on zero, 15, 30, 60 and 120 days after nanoparticles preparation. Our results showed that the follow up of size and zeta potential (up to 120 days) revealed the stability of prepared M-CS/Le NPs. The size, on all measured times, was in the nano scale (less than 100 nm). Where, TEM image showed that M-CS/Le NPs has a size of 97 nm after melatonin loading (on 0 day after synthesis). The hydrodynamic size of M-CS/Le NPs (93.2 nm), obtained by DLS, was similar to that obtained from TEM (97 nm). The size of nanoparticles started to slightly increase on the 15 days post synthesis; then, stayed constant up to 120 days post synthesis. The zeta potential was higher than 30 mV indicating the stability of M-CS/Le NPs. On the 0 day after synthesis, CS/Le NPs has a positive zeta potential (25 mV) that increased to 32.8 mV after melatonin loading. The zeta potential of CS/Le NPs increased to 27 mV, on the 15 days post synthesis, then stayed unchanged up to 120 days. On the other hand, the zeta potential of M-CS/Le NPs increased to 34.2 mV and remained constant up to 120 days. A controlled and sustained release of melatonin was observed up to 48 h. The M-CS/Le NPs have a high antimicrobial activity against *Bacillus subtilis*, *Staphylococcus aureus*, *Escherichia coli*, *Pseudomonas aeruginosa*, *Candida albicans* and *Mucor*. It, also, showed powerful anti-inflammatory (hemolysis inhibition %), antioxidant (scavenged DPPH, H_2_O_2_, OH^−^ and O^2−^) activities and a negligible cytotoxicity (high cell viability % in MTT assay).

Our results were in agreement with those of Hafner et al. (2009) who investigated M-CS/Le NPs prepared from different lecithin types (Lipoid S100, S75 and S45) with different chitosan/lecithin ratios. They found that M-CS/Le NPs have a size of 121.6 to 347.5 nm and a zeta potential between 7.5 and 32.7 mV. They reported that both of size and zeta potential increased with increasing the lecithin amount in the preparation. On the other hand, Lopes Rocha Correa et al. (2020) prepared M-CS/Le NPs by chitosan/lecithin ratio of 1:1 and found an EE% of 27%. M-CS-Le NPs have a size of 160.43 nm and a positive zeta potential of 25 mV.

The animal model in this study was performed with male Sprague Dawley rats. The induction of diabetes was done by the intravenous injection of STZ (65 mg/kg) according to Furman (2015). In this study, diabetes induction was confirmed, along all experimental periods, by measuring the levels of FBG and FTA in diabetic groups (GI to GIX) in comparison to those of their corresponding normal non-diabetic groups (GI to GIX). It was obvious that a significant increase in FBG and FTA levels (more than 280 mg/dl and 240 µmol/l, respectively) was noticed in diabetic groups when compared to those of non-diabetic groups (125–130 mg/dl and 125–138 µmol/l, respectively).

After diabetes induction, full thickness-induced wounds was created on the rats’ back after fur shaving and cleaning with 70% ethanol. *Staphylococcus aureus* was chosen for infection of the induced wounds in this study. *Staphylococcus aureus* is a prevalent infectious agent that present a high risk of infection in fifty to sixty% of non-healing wounds. When the skin is injured, pathogenic bacteria can penetrate the underlying tissue, which sets off a complex physiological reaction that involves the migration of immune cells to the site of infection (Kobayashi et al. 2015).The most frequent pathogen identified from diabetic skin lesions was *Staphylococcus aureus* (Thurlow et al. 2020). Furthermore, in diabetic individuals, *Staphylococcus aureus*-infected wounds are more likely to develop in invasive infections such as endocarditis, osteomyelitis, and sepsis (Smit et al. 2016; Jneid et al. 2018). Several virulence factors characterize *Staphylococcus aureus* capacity to harm diabetic wounds, with released toxins playing a key role (participation in colonization, persistence, immune system evasion, and spread) (Vandenesch et al. 2012). These toxins include toxins of superantigens, pore forming and epidermal cell differentiation inhibitor; in addition to, exfoliatin. These cytolytic toxins can disrupt host cell membranes, resulting in cell lysis (Grumann et al. 2014). Red blood cells are lysed by hemolysins, while white blood cells are targeted by leukotoxins.

Because it might be challenging to control recurrent inflammatory reactions in these wounds, chronic wound treatment remains a challenge. One reason for this is the growth of bacterial biofilms inside and on the surface of chronic wounds. TNF-α and IL-6 are two examples of the inflammatory cytokines that accumulate when neutrophils and macrophages are activated by bacterial biofilms (Wu et al. 2019). On the other hand, the immune milieu that is dysregulated in chronic wounds encourages the growth of bacteria, which sets off a cycle of biofilm formation and persistent inflammatory reactions. Because bacterial biofilms rapidly develop antibiotic resistance, have a high microbial species diversity, prevent the penetration of the antimicrobial agents, and present a number of other problems, treating biofilms therapeutically can be difficult (Omar et al. 2017).

In this study, after wound induction, animals received topical administration of free melatonin, CS/Le NPs and M-CS/Le NPs twice a day (one every 12 h) for 14 successive days. The chosen dose of M-CS/Le NPs was 5 mg/kg that is half the used dose of free melatonin (10 mg/kg). This was designed to examine the possibility that nanoparticles can help in the reduction of melatonin dose. The wound area % was calculated on the 1st, 7th and 14th days after wound induction. From each group, three rats were anesthetized with sodium pentobarbital on the 1st, 7th and 14th days after wound induction in order to collect skin samples for measurements of oxidative stress and immunological parameters.

On the 1st day PT, wound induction significantly raised MDA and decreased SOD levels in normal non-diabetic animals. Wound infection dramatically increased the levels of oxidative stress and disturbed the antioxidant enzyme levels. It was clear that introduction of diabetes led to a considerable increase in the MDA levels in the diabetic groups compared to the normal non-diabetic ones. In diabetic GVI animals, with untreated infected wounds, the highest MDA and lowest SOD levels were observed. Pro-inflammatory cytokines (TNF-α and IL-1β) were found to be much higher in the skin tissue homogenates of diabetic groups than in non-diabetic groups. Cytokine levels were greater in the groups with infected wounds than in the uninfected wound groups.

On the 7th day PT, the rise in MDA and the fall in SOD levels proceeded to reach their maximum peak for the entire study period. In every group, the maximum levels of cytokines were noted. Furthermore, out of all the experimental groups, diabetic GVI (with untreated infected wounds) had the greatest levels of cytokines.

On the 14th day PT, treatment of wounds with CS/Le NPs or free melatonin alone was unable to return the MDA and SOD levels to normal, comparable to normal control GI. In treated non-diabetic or diabetic groups (GV and GIX), M-CS/Le NPs administration effectively restored the MDA and SOD levels to those of their respective control groups. The cytokine levels in the non-diabetic groups (GV and GIX) treated with M-CS/Le NPs were comparable to those in the healthy control GI. Following treatment with M-CS/Le NPs, diabetic groups with both uninfected (GV) and infected (GIX) wounds displayed cytokine levels similar to those of diabetic control GI.

Alongside this, there was a notable rise in the pro-apoptotic proteins (Bax, p53, casapase3, and 8) and a fall in the anti-apoptotic protein Bcl2. In both non-diabetic and diabetic untreated infected groups (N GVI and D GVI), previous disturbance (caused by wound induction) was noticeably higher. The increased levels of apoptosis and necrosis in skin cells were somewhat reduced by the administration of free melatonin or CS/Le NPs, but these treatments had no impact on the infected diabetic groups. Skin cell apoptosis was dramatically reduced by the administration of M-CS/Le NPs, particularly in the infected diabetic groups. M-CS/Le NPs successfully aided in the wound healing process and decreased the wound area % in healthy and diabetic treated groups more than free melatonin or CS/Le NPs.

The positive surface charge of prepared M-CS/Le NPs enhanced the wound healing in diabetic rats, where the positively charged nanoparticles adhered to the cells in the wound area. Venault et al. (2019) showed that positively charged hydrogel accelerated the wound healing mechanism by increasing cell proliferation and maturation more than negatively charged one. Huang et al. (2018) and Lopes Rocha Correa et al. (2020) added that based on scanning electron microscope (SEM) images, M-CS/Le NPs was surrounded by a layer of free chitosan. This may be due to the macroion-polyion interactions between the chitosan and lecithin (Gerelli et al. 2008). Moreover, the antibacterial effect of M-CS/Le NPs can be attributed to the surface interaction of nanoparticles with teichoic acid (in the cell wall of *Staphylococcus aureus*) leading to cell membrane extraction. Shi et al. (2016) reported that the bacterial membranes were impaired due to the charge interaction between cationic nanoparticles and bacteria. Upadya et al. (2011) showed that the antibacterial activity of chitosan nanoparticles can be attributed to their nanosize that provided an increased surface area for attachment with bacterial cell membrane. Cobrado et al. (2012) added that this attachment of nanoparticles to bacterial surface inhibited the biofilm formation by inhibiting the protein synthesis and, hence, the bacterial growth. Hafner et al. (2011) showed that part of the melatonin released from M-CS/Le NPs penetrate the skin cells and another part remained on the skin surface. However, Buscemi et al. (2006) and Priano et al. (2007) added that melatonin has a limited adverse effects even when received at high dose. Moreover, several studies reported the wound healing effect of melatonin loaded nanoparticles. Blažević et al. (2016) showed a decrease in wound area % in wounds treated with M-CS/Le NPs. Lopes Rocha Correa et al. (2020) reported that groups treated with M-CS/Le NPs showed a significant reduction in wound area % more than those treated with unloaded CS/Le NPs or free melatonin.

Our results can be explained by the medicinal properties of melatonin, chitosan and lecithin. According to Mawazi et al. (2024), chitosan has a well-established bactericidal action, especially against two important pathogens (*Pseudomonas aeruginosa* and *Staphylococcus aureus*) in diabetic wounds. Chitosan speeds up wound closure by promoting cell proliferation, the accumulation of collagen, and re-epithelialization (Rajinikanth et al. 2024). By facilitating the electrostatic interactions with negatively charged molecules on the bacterial cell membrane, the positively charged CS/Le NPs impairs the integrity of the membrane and prevents bacterial development (Yan et al. 2021). This implies that CS/Le NPs can maintain their antibacterial properties and stop subsequent infections even after melatonin release. Even after the release of melatonin, the remaining CS/Le NPs may influence immune responses by lowering oxidative stress and pro-inflammatory cytokines, which aids in wound healing. The emollient and skin barrier-repairing properties of lecithin are well established. Therefore, lecithin in CS/Le NPs may improve hydration, prevent the oxidative damage, and encourage epithelial cell migration even after melatonin release from the nanoparticles.

In conclusion, M-CS/Le NPs promoted the wound healing in *Staphylococcus aureus*-infected wounds in diabetic rats. This remarkable wound healing effect can be attributed to: 1- the antioxidant and anti-inflammatory properties of melatonin (Reiter et al. 2000; Rodríguez et al. 2007), 2- the combined antibacterial effect of chitosan (Cobrado et al. 2012) and melatonin that showed antibacterial activities against gram-negative, gram-positive and *Mycobacterium tuberculosis* (He et al. 2021) and 3- the reduction in oxidative stress and inflammatory markers that created a suitable environment for skin cell maturation (de Souza et al., 2020). Moreover, the preparation of M-CS/Le NPs decreased the dose of used melatonin (when compared to free melatonin). The study has some limitation like the examination of single dose of M-CS/Le NPs on a small number of animals. Also, the lack of CFU quantification in wound tissues limited the direct assessment of bacterial clearance following treatment. However, further studies are required to examine the wound healing effect of lower doses of M-CS/Le NPs (than 5 mg/kg). While acetate buffer was the most suitable choice for the release study, additional release studies in other physiological conditions (e.g., PBS, enzyme-containing media, or simulated wound fluid) would provide a broader understanding of M-CS/Le NPs’ behavior in different environments; in addition to accelerated stability test where nanoparticles are subjected to higher temperatures (e.g., 25 and 40 °C) and humidity-controlled conditions for a defined period. Future studies could investigate the effect of buffer composition, pH variations, and enzyme presence on melatonin release to enhance the clinical translation. Further studies on the systematic in vivo toxicity evaluation (long-term effects on liver/kidney function, immune response) are recommended. Moreover, comparing M-CS/Le NPs with nano-ionic formulations in wound healing is recommended to determine the drug stability, bioavailability, and wound healing efficacy. Extended biocompatibility testing in human-derived skin cell models can be a step towards clinical evaluation.

## Data Availability

The data generated or analysed during this study will be available on a reasonable request.
